# Regular exercise delays microvascular endothelial dysfunction by regulating antioxidant capacity and cellular metabolism

**DOI:** 10.1038/s41598-023-44928-4

**Published:** 2023-10-17

**Authors:** Giorgia Scarfò, Simona Daniele, Elisa Chelucci, Antonio Rizza, Jonathan Fusi, Giancarlo Freggia, Barbara Costa, Sabrina Taliani, Paolo Artini, Claudia Martini, Ferdinando Franzoni

**Affiliations:** 1 Division of General Medicine, Department of Clinical and Experimental Medicine, University of Pisa, Pisa, Italy; 2 Department of Pharmacy, University of Pisa, Pisa, Italy; 3 Interventional Cardiology Division, Gaetano Pasquinucci Heart Hospital, Fondazione Toscana Gabriele Monasterio, 54100 Massa, Italy; 4 Cardiology Unit, Gaetano Pasquinucci Heart Hospital, Fondazione Toscana Gabriele Monasterio, 54100 Massa, Italy; 5 Division of Gynecology and Obstetrics, Department of Clinical and Experimental Medicine, University of Pisa, Pisa, Italy

**Keywords:** Biochemistry, Molecular medicine, Cardiovascular diseases, Metabolic disorders

## Abstract

Aging is the basis for several unfavorable conditions, including cardiovascular diseases (CVDs). In this sense, regular physical activity (regular PA) has been proven to delay cellular aging and prevent endothelial dysfunction related to CVDs. Despite numerous studies involving athletes, little is known about cellular and molecular mechanisms of regular PA among master athletes. The present study aimed at evaluating the effects of regular PA on local microcirculatory functions in elderly athletes as compared to age-matched sedentary controls. Moreover, molecular/epigenetic mechanisms (nitric oxide, oxidative stress, PGC-1α, SIRT1 and miR29) were also assessed. The results of the present study showed that regular PA significantly increased local blood flow in post-ischemia and post-heating conditions, as well as NO plasma concentrations, denoting a better endothelial function/microcirculatory efficiency. Moreover, athletes presented a greater plasma antioxidant and increased transcriptional levels of the metabolism regulator PGC-1α. Finally, regular PA enhanced plasma level of SIRT1 and miR29, suggested as epigenetic regulators of redox balance and cellular metabolism. In addition, stimulated local blood flow was directly related to plasma antioxidant capacity, and SIRT1 and miR29 levels. Overall, our data confirm the beneficial effects of regular PA on the cardiovascular profile in elderly athletes and shed light on molecular signals involved in the positive adaptations to exercise.

## Introduction

Aging is a physiopathological process characterized by a gradual structural and functional decline of the organism over time and lays the basis for several unfavorable conditions including cardiovascular diseases (CVDs)^1^. Although the molecular mechanisms underlying aging are still not entirely clear, the process seems to be associated with homeostatic disorders, mitochondrial dysfunction, genetic factors, epigenetic modifications, telomere length, and impaired intracellular signaling pathways^2^.

From a cardiovascular point of view, arteriosclerosis and endothelial dysfunction are the greatest manifestations of vascular aging. In particular, the first one consists in a progressive arterial stiffness and atherosclerotic processes with lipid oxidation, inflammation, and arterial plaques^1^; similarly, endothelial dysfunction is defined by an impaired vascular reactivity, but it also involves proinflammatory and prothrombotic state and may contribute also to the development, progression, and clinical complications of atherosclerosis^3,4^. These mechanisms have been associated with a higher risk of hypertension, coronary artery diseases, cardiac remodeling, and fibrosis which in turn could cause arrhythmias and heart failure^5^. Specifically, with advancing age, endothelial cells undergo cell cycle dysregulation, which is primarily due to a decreased NO bioavailability, as well as inflammatory states^6^. Indeed, NO, a free radical produced during several physiological processes, is mostly involved in the regulation of the vascular tone by inducing the relaxation of vascular smooth muscle cells^7^. The main source of NO within human organisms is the nitric oxide synthase (NOS) family, whose endothelial expression tends to decrease with aging^8^. In addition to the decreased bioavailability of NO, redox imbalance is a crucial factor of cell senescence: oxidative stress, deriving from an imbalance between the antioxidant defenses and the uncontrolled production of reactive oxygen and nitrogen species (RONS), impairs lipids, proteins, and nucleic acids, and is responsible for cell damage and the consequent endothelial dysfunction^9^.

Although the above-mentioned mechanisms are completely avoidable, exercise stands as the best way to delay these aging-related processes^10^. It has been widely demonstrated that regular physical exercise (regular PA) is able to counteract inflammation and the negative effects of free radicals by inducing an adaptive response in endogenous antioxidant systems^11^. Generally, in athletes, levels of catalase (CAT) and glutathione peroxidase (GPx), are increased; in contrast, lipid peroxidation, which is considered the main oxidative marker, is considerably decreased^12^. Strenuous exercise was shown to cause immediate and delayed alterations in cardiovascular function^13,14^, however, the cardiovascular health benefits of acute and regular aerobic exercise are irrefutable^15,16^. Exercise has also been proven to affect positively endothelial functions thanks to its capacity of increasing NO bioavailability in both young and older subjects^17^, since physically active old individuals have demonstrated preserved microcirculatory functions and plasma antioxidant defenses in the cutaneous microcirculation^18^. Moreover, exercise has been associated with a better vascular tone and response to external factors demonstrating an excellent endothelial cell response^10^.

Recent literature is shedding light on the molecular mechanisms underlying the protective effects of regular PA. For example, exercise has been proven to involve the AMPK/SIRT1/PGC-1α signaling cascade, thereby regulating mitochondrial biogenesis and oxidative stress within cells^19^. Specifically, the transcriptional co-activator PPAR-γ co-activator-1 α (PGC1-α) plays a fundamental role in mitochondrial biogenesis and affects glucose-fatty acid metabolism: particularly, the expression of PGC1-α is higher in tissues with a great presence of mitochondria and oxidative pathways, including brain and skeletal muscles^20^. During exercise, activation, and production of AMPK are positively correlated with ROS accumulation in skeletal muscles, contributing to triggering the AMPK/SIRT1/PGC-1α signaling pathway.

Epigenetics can be considered as the connection between genetic determinants predisposing to CVDs and environmental factors, including exercise^1^. Indeed, epigenetics is necessary for the maintenance of tissue-specific gene expression development, cardiovascular homeostasis, and gene plasticity changes^2^ and, besides the most common risk factors for CVDs such as diabetes, obesity, smoking^21^, vascular aging is affected by epigenetic mechanisms too. For example, SIRT1, a member of the nicotinamide adenine dinucleotide (NAD)-dependent histone deacetylases Sirtuins, which have been demonstrated to play a key role in regulating the antioxidant redox signaling in response to regular PA too^22^. In addition, non-coding RNA, microRNA (miRNA), and long non-coding RNA have been suggested to exert a key role in endothelial senescence-related gene expression^2^. In this sense, regular PA is able to regulate circulating cardiovascular-related miRNAs with remarkable physiological consequences^23^.

Besides this evidence, the molecular mechanisms underlying the protective effects of regular PA on cardiovascular systems in elderly are still ongoing. Further understanding of the role of epigenetics in aging processes may provide additional insights into unveiling age-related CVDs with the ultimate goal of developing new strategies for treating them.

The aim of the present study was to evaluate the long-term effects of regular PA on plasma antioxidant capacity and microcirculatory functions in a population of master athletes. Moreover, molecular and epigenetic mechanisms were considered to understand the effects mediated by exercise.

## Results

Results showed that regular exercise could prevent aging-related endothelial dysfunction by improving and preserving plasma antioxidant defenses in the cutaneous microcirculation through the regulation of cellular pathways involved in redox signaling.

### Descriptive statistics

As shown in Table 1, no significant differences in age and body mass index (BMI) were found between ATL and SED. Athletes had a lower basal heart rate (56 + 7 bpm in ATL vs. 70 + 11 bpm in SED) and higher VO2 max (58 ± 7 ml/kg/min in ATL vs 43 ± 6 ml/kg/min in SED, *p* < 0.01) than sedentary controls.

**Table 1:** Descriptive statistics of selected parameters for ATL and SED.

Parameter	Athletes (mean ± SD)	Sedentary (mean ± SD)	*p* value
Age (years)	52.39 ± 15.31	51.40 ± 14.83	> 0.05
BMI (Kg/m^2^)	22.19 ± 1.21	23.09 ± 1.55	> 0.05
VO2 max (ml/Kg/min)	58 ± 7	43 ± 6	< 0.01
NOx (mM)	50.35 ± 18.97 **	38.99 ± 15.27	< 0.01
HBF basal (pu)	206.33 ± 34.93	189.59 ± 37.65	> 0.05
HBF heating (pu)	332.04 ± 36.36 ***	270.50 ± 35.36	< 0.001
HBF ischemia (pu)	374.42 ± 38.43 ***	277.64 ± 42.16	< 0.001
FBF basal (pu)	206.78 ± 34.39	184.50 ± 36.26	> 0.05
FBF heating (pu)	225.08 ± 43.27 ***	179.45 ± 38.73	< 0.001
FBF ischemia (pu)	378.46 ± 37.74 ***	269.23 ± 40.86	< 0.001
SIRT1 (ng/ml, plasma)	4.05 ± 0.613 ***	2.84 ± 0.80	< 0.001
miR29	4.25 ± 0.73 ***	3.07 ± 0.65	< 0.001
PGC-1α mRNA	2.73 ± 0.70 ***	1.89 ± 0.65	< 0.001
TOSC vs. peroxyl radicals	18.74 ± 2.62 ***	12.01 ± 1.91	< 0.001
TOSC vs. hydroxyl radicals	9.86 ± 1.50 ***	6.04 ± 1.50	< 0.001

The data are expressed as mean ± SD. Statistical analysis was performed by unpaired t-tests: **p < 0.01, ***p < 0.001 (ATL vs. SED). *BMI* body mass index; *VO2 Max* maximal oxygen consumption, *NOx* nitric oxide, *HBF* hand blood flow, *FBF* foot blood flow, *IRT1* Sirtuin 1, *PGC-1α* PPAR-γ co-activator-1 α, *TOSC* total oxyradical scavenging capacity.

### Clinical and biochemical parameters in ATL and SED subjects

**Figure 1:**
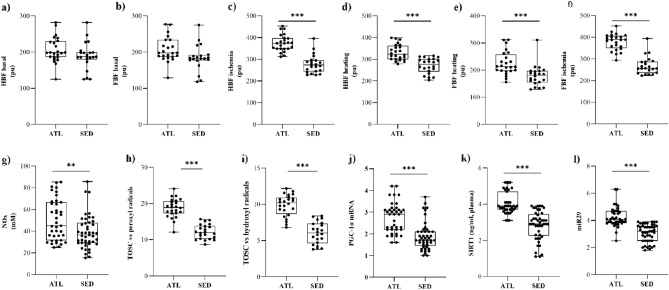
Descriptive statistics of selected parameters for ATL and SED. The data are reported as mean ± SD. Statistical analysis was performed by unpaired t-tests: **p < 0.01, ***p < 0.001 (ATL vs. SED). HBF (Hand Blood Flow); FBF (Foot Blood Flow); NOx (Nitric Oxide); TOSC (Total Oxyradical Scavenging Capacity); PGC-1α (PPAR-γ co-activator-1 α); SIRT1 (Sirtuin 1).

At rest, skin microcirculation was similar between the two groups, as demonstrated by basal HBF (Fig. 1a) and FBF (Fig. 1b) which did not differ significantly. In ATL, HBF significantly increased in response to heating and ischemia by 164.05 ± 24.69% (*P* = 0.0003) and 186.26 ± 34.97% (*P* < 0.0001), respectively. In SED, the enhancement of HBF was only 146.28 ± 26.29% (*P* = 0.0126) post-heating, and 151.15 ± 36.33% (*P* = 0.0059) post-ischemia. FBF post-heating increased in ATL by 110.79 ± 32.20% (*P* ≥ 0.05) and post-ischemia by 189.31 ± 40.32% (*P* < 0.0001), respectively. In contrast, in SED subjects FBF increased only in response to ischemia by 147.39 ± 27.17% (*P* = 0.0012). HBF post-ischemia and post-heating was significantly higher in ATL than in SED (*P* < 0.001 for all the measurements; Fig. 1c,d), demonstrating improved vessel reactivity given by an excellent endothelial function. FBF (post-heating and post-ischemia) was significantly higher in ATL than in SED (*P* < 0.001 for all the measurements; Fig. 1e,f). NO plasma concentrations were greater in ATL indicating a preserved bioavailability of NO despite the advancing age (*P* < 0.01; Fig. 1g).

Next, biochemical determinations were performed on plasma and blood cell samples of the same subjects, to unveil specific signals related to oxidative stress and its epigenetic mechanisms of regulation. Both TOSC values vs ROO· and OH·, index of the plasma antioxidant capacity, were higher in ATL than in SED (*P* < 0.001 for all the measurements; Fig. 1h,i), thus denoting that ATL exhibited higher antioxidant activities in plasma.

Moreover, the mRNA levels of PGC-1α, chosen as a marker of metabolism related to oxidative stress^24^, were significantly elevated in ATL than in SED subjects (*P* < 0.001; Fig. 1j).

SIRT1 and miR29 were chosen as linked to PGC-1α and oxidative stress^16^. The results of the present study indicated that ATL presented significantly higher plasma levels of SIRT1 (*P* < 0.001; Fig. 1k) and miR29 (*P* < 0.001; Fig. 1l) than SED subjects.

### Correlations between clinical parameters

Correlations between clinical parameters are shown in Table 2 and Supplementary Table S1. NOx concentration positively correlates with both HBF post-heating (*P* < 0.05; Table 2) and post-ischemia (*P* < 0.05; Table 2), and with FBF post-ischemia (*P* = 0.0011; Table 2). HBF post-heating was positively related to post-heating FBF (*P* = 0.0005; Table 2). Similarly, HBF post-ischemia positively correlates with FBF post-ischemia (*P* < 0.0001; Table 2).

**Table 2:** Correlation between clinical parameters; p values obtained for each correlation are reported in the respective columns.

	Correlation	*p* value
NOx (mM), HBF heating	0.300	0.0424
NOx (mM), HBF ischemia	0.312	0.0345
NOx (mM), FBF ischemia	0.459	0.0011
HBF heating, FBF heating	0.487	0.0005
HBF ischemia, FBF ischemia	0.823	< 0.0001

*NOx* nitric oxide, *HBF* hand blood flow, *FBF* foot blood flow.

### Correlations between biochemical parameters

All biochemical parameters were correlated by linear regression analysis (Table 3). SIRT1 was found to positively correlate with TOSC versus ROO· (*P* = 0.0039; Table 3), TOSC versus OH· (*P* = 0.0041; Table 3), miR29 (*P* < 0.0001; Table 3) and PGC-1α mRNA (*P* = 0.0063; Table 3). Similarly, miR29 and PGC-1α mRNA were positively related to TOSC values versus ROO· (*P* = 0.0001 and *P* = 0.0009, respectively; Table 3) and versus OH· (*P* < 0.0001 and *P* = 0.0084, respectively; Table 3).

**Table 3:** Correlation between biochemical parameters; *p* values obtained for each correlation are reported in the respective columns.

	Correlation	*p* value
SIRT1 (ng/ml, plasma), miR29	0.425	< 0.0001
SIRT1 (ng/ml, plasma), PGC-1α mRNA	0.298	0.0063
SIRT1 (ng/ml, plasma), TOSC vs. peroxyl radicals	0.422	0.0039
SIRT1 (ng/ml, plasma), TOSC vs. hydroxyl radicals	0.416	0.0041
miR29, TOSC vs. peroxyl radicals	0.528	0.0001
miR29, TOSC vs. hydroxyl radicals	0.545	< 0.0001
PGC-1α mRNA, TOSC vs. peroxyl radicals	0.473	0.0009
PGC-1α mRNA, TOSC vs. hydroxyl radicals	0.381	0.0084

*SIRT1* Sirtuin 1, *PGC-1α* PPAR-γ co-activator-1 α, *TOSC* total oxyradical scavenging capacity.

### Correlation between clinical and biochemical parameters

All clinical parameters were correlated with biochemical ones by linear regression analysis (Supplementary Table S2). NOx concentrations were positively related to miR29 (*P* = 0.0484) and to TOSC values versus ROO· (*P* = 0.0030) and OH· (*P* = 0.0021).

Interestingly, SIRT1 was directly correlated with basal HBF (*P* = 0.0265), HBF post-heating (*P* = 0.0052), HBF post-ischemia (*P* = 0.0019), basal FBF (*P* = 0.0148), FBF post-heating (*P* = 0.0030), and FBF post-ischemia (*P* = 0.003).

Similarly, TOSC values were found to be related positively to: basal FBF (TOSC vs. ROO·: *P* = 0.0461; TOSC vs. OH·: *P* = 0.0435); HBF post-ischemia (*P* < 0.0001 in both cases); FBF post-heating (TOSC vs. ROO·: *P* = 0.0005; TOSC vs. OH·: *P* = 0.0013); FBF post-ischemia (TOSC vs. ROO: *P* ≤ 0.0001; TOSC vs. OH·: *P* ≤ 0.0001); and HBF heating (*P* < 0.0001 in both cases).

Finally, miR29 was positively related to: HBF post-ischemia (*P* ≤ 0.0001); FBF post-heating (*P* = 0.0108); FBF post-ischemia (*P* = 0.0009); and HBF post-heating (*P* = 0.0141).

## Discussion

The main findings of the present study are the following: (1) HBF (post-ischemia and post-heating) and NO plasma concentrations were significantly higher in ATL than in SED, overall denoting a better endothelial function and microcirculatory efficiency; (2) ATL presented a greater plasma antioxidant capacity against both hydroxyl and peroxyl radicals, and increased transcriptional levels of the metabolism regulator PGC-1α; (3) ATL presented higher levels of SIRT1 and miR29, suggested as epigenetic regulators of redox balance and cellular metabolism. In addition, we found that: (1) HBF and FBF positively correlated with NOx concentration, plasma antioxidant capacity, and plasma levels of SIRT1 and miR29; (2) plasma antioxidant capacity was positively correlated with PGC-1α mRNA, SIRT1, and miR29 levels. Overall, the above data confirm the beneficial effects of regular PA on the cardiovascular system and the molecular and intracellular positive adaptations to exercise.

CVDs are the world’s leading cause of death; aging could be considered the most predisposing factor for these pathologies, being associated with structural and functional abnormalities affecting heart and blood vessels^25^. These macroscopic alterations are accompanied by the impairment of intracellular signaling pathways, including those related to inflammation and oxidative stress^26^, with the consequent promotion of cell senescence. In this sense, regular PA, particularly aerobic exercise, has proven to positively affect cell redox state by improving the activity of enzymatic and non-enzymatic antioxidant defenses systems^27^, finally delaying cellular senescence^28^.

In our hands, ATL had a lower basal heart rate and higher VO2 max than SED, as reported previously (58 ± 7 ml/kg/min in ATL vs. 43 ± 6 ml/kg/min in SED, *p* < 0.01). Basal blood flow at rest was comparable in the two groups. Nevertheless, ATL had better microcirculation, as demonstrated by the greater HBF and FBF post-ischemia or post-heating. Therefore, exercise has been demonstrated to induce physiological adaptations in the cardiovascular system, such as a decrease in total peripheral resistance associated with an increase in cardiac output in order to ensure better perfusion of skeletal muscles and organs involved in supporting exercise as well as to provide an adequate thermal dispersion^29^. As previously demonstrated, vasodilation of peripheral arterioles and skin microcirculation are markers of an efficient endothelium-dependent vessel release that reflects a good and preserved endothelial function^29,30^.

In ATL, the increase in HBF and FBF post-heating and post-ischemia conditions reflects the capacity of blood vessels to be more responsive under conditions in which oxygen demand increases or there is a need for thermal dispersion, as occurs during PA. Moreover, as demonstrated by the fact that basal skin blood flow did not differ significantly between the two groups, it could be argued that rather than improving endothelial function under normal conditions exercise improves the responsiveness of the endothelium to particular circumstances, consistent with previous studies^29,31,32^.

The improvement in endothelial function is often supported by changes that occur in the bioavailability of bioactive compounds^29^. In our hands, the enhancement in microcirculation evidenced in master athletes can be explained, at least in part, by the increased bioavailability of NO, as demonstrated by greater NO plasma concentrations in ATL. Indeed, advancing age is related to a decrease in NO bioavailability, due to a diminished eNOS expression, which is required for NOS-dependent production of NO, as well as to increase activity of arginase, able to impair NOS function^33^. Moreover, mild regular aerobic-endurance exercise or combined aerobic and low-intensity resistance exercise training are sufficient to increase NO production in previously sedentary older humans^34,35^, probably by an increase of eNOS expression^17,36,37^. Of note, skin vasodilation depends mainly on NO production during endurance exercise^13,38^, with a positive correlation between stimulated blood flow and NO concentrations.

Next, biochemical determinations were performed on plasma and blood cell samples of the same subjects, to unveil specific signals related to oxidative stress and its epigenetic mechanisms of regulation. Plasma antioxidant activities toward hydroxyl and peroxyl radicals were significantly higher in ATL subjects as compared to SED ones, thus confirming our previous report^12,39–41^. Interestingly, TOSC values toward hydroxyl and peroxyl radicals were found to be directly related to NOx concentrations and blood flow post-heating and post- ischemia. The increased NO bioavailability, accompanied by an enhanced antioxidant capacity, mirrors evidence showing that oxidative stress causes the oxidation of NOS cofactor BH4, an excess of endogenous methylarginines, and a lack of l-arginine, thereby leading to eNOS uncoupling. Therefore, ROS diminishes NO amount at the endothelial level^42^. In this sense, better antioxidant defenses prevent endothelial cell damage caused by oxidative stress, preserving the endothelial function and the endothelium-dependent vasodilatation of blood vessels^43^. Overall, these findings confirmed that an efficient redox balance with an improvement of the antioxidant responses predisposes to an enhancement in the microcirculatory system. In considering our results, it should be mentioned that emerging evidence supports that regular strenuous exercise increases coronary artery disease risk^44^ and oxidative stress in the tunic media^45,46^.

Furthermore, molecular mechanisms were considered to understand the effects mediated by exercise. In our hands, ATL presented significantly higher blood levels of mRNA levels of PGC-1α. PGC1-α plays a crucial role in exercise-induced adaptations to endurance training since it exerts beneficial effects on mitochondrial biogenesis, angiogenesis^47^ and heart function^48^. More generally, PGC1-α levels can mirror the cardiorespiratory fitness and metabolic conditions of the organism, since higher levels indicate a prevalent oxidative metabolism and better antioxidant defenses; in contrast, when levels of PGC-1α are decreased, redox balance is impaired, with a consequent ROS overproduction^19^. Consistent with this strict connection between ROS and PGC-1α, herein we found a positive correlation between PGC-1α mRNA and plasma antioxidant capacity versus hydroxyl and peroxyl radicals.

Epigenetics could explain how environmental and lifestyle factors, including exercise, are able to affect organism physiopathological processes and genetic determinants: in this regard, the histone deacetylase SIRT1 exerts a primary role in determining exercise-induced cellular adaptations triggering several cellular pathways that regulate inflammation and oxidative stress^49^. In our hands, ATL presented a higher plasma concentration of SIRT1, thus confirming that regular exercise is able to trigger SIRT signal. In addition, SIRT1 directly correlated with local blood flow at rest and post-heating. Our findings are consistent with the strict connection between SIRT1 and intracellular signaling pathways involved in redox homeostasis, leading to the improvement of the endothelial-dependent relaxation^50^.

Moreover, SIRT1 levels were also found to be related to plasma antioxidant capacity and PGC-1α mRNA. Consistently, the interconnection of ROS, PGC-1α has been suggested to be related to AMPK-SIRT1 during exercise^19,48,51,52^, confirming that SIRT1 plays a pivotal role in mediating the adaptation in metabolism and antioxidant responses related to regular physical exercise.

Finally, ATL were demonstrated to present higher levels of plasma miRNA29, selected as one of the miRNAs altered in cardiovascular disease and possibly related to regular physical exercise^23^. The miRNA concentration was related positively to the subjects’ plasma antioxidant capacity, thereby confirming the strict connection between miRNA concentration and oxidative stress^53^. However, in a previous study, miR-29b expression has proven to be negatively correlated with ROS levels negatively affecting SIRT1 activity in tumoral ovarian cells^54^, even though these differences may be related to the tumor microenvironment that has been considered in this paper.

## Materials and methods

### Subjects’ enrollment

36 competitive athletes, long-distance sex-matched runners (ATL, age range: 47–74 years) recruited by the “Marathon Club Pisa,” and 36 healthy sedentary sex- matched volunteers (SED, age range: 46–77 years) were studied. Subjects were selected at the University of Pisa, specifically, athletes during the annual preparticipation screening, while healthy volunteers were selected among those who underwent cardiological examination.

Subjects were enrolled in absence of previous CVDs or risk factors, evaluated through an accurate medical history and clinical examination which comprised the analysis of anthropometric parameters, baseline electrocardiogram, echocardiogram. Subjects with coronary artery disease, previous myocardial infarction or stroke, evidence of cardiomyopathy were excluded. In addition, subjects with at least one of the following risk factors were excluded: smoking, alcohol, diabetes, hypertension, dyslipidemia. A COSMED system cardiopulmonary exercise testing was used to assess maximal oxygen consumption (VO2 max). All subjects performed a maximal test on a cycle ergometer using a one-minute incremental test (25 W/minute). Those who performed vigorous endurance exercise (> 5 times/week) for at least 10 years and with VO2 max > 50 ml/kg/min^55^ were considered athletes. Subsequently, subjects in both groups underwent laser Doppler flowmetry to study skin microcirculation, and blood sampling to determine plasma antioxidant capacity after a period of rest from PA for at least one day. The study was performed following the guidelines of the Declaration of Helsinki and agreed by the Ethics Committee (CTO, Clinical Trial Office) of Azienda Ospedaliero Universitaria Pisana (AOUP) (protocol code 35,105, approved on 13 June 2019).

### Microcirculatory study

Subjects underwent a laser Doppler study of the skin blood flow (SBF). A laser Doppler flowmetry (Periflux PF4001, standard probe PF408; Perimed, Jarfalla, Sweden) in the upper and lower extremities was used in order to detect both hand blood flow (HBF) and foot blood Flow (FBF) as an index of endothelial function and microcirculatory efficiency^17^. The laser Doppler probe was attached to the skin surface of the third finger of the left hand and to the first toe of the left foot. HBF and FBF were measured under basal conditions, after local heating to 44 °C, and during reactive hyperemia following a 3 min brachial artery occlusion with the aim of evaluating microcirculation under hyperemia and hypoxia conditions. The output signal was linearly related to red blood cell flow as predicted by theoretical^56^ and experimental^57^ models. The laser Doppler apparatus was connected to a PC via RS232 interface. The program installed on the computer (Perisoft) allows storage and analysis of the recordings. The measurement of SBF is expressed in arbitrary units, “perfusion units” (pu). The coefficient of variation (three measurements) for basal SBF was less than 5%.

### Blood sample, mononuclear cells, and plasma

All subjects were examined at 9 a.m. in a quiet, air-conditioned room with temperature maintained at 22–24 °C. After the introduction of an Indwelling cannula (Abbocath 20 G) into the left cephalic vein, each subject was allowed to rest in a supine position for at least 15 min before monitoring blood pressure. Venous blood samples were collected in tubes containing dipotassium ethylenediaminetetraacetic acid (EDTA) and immediately processed for plasma separation.

Ficoll-Paque™ (Miltenyi Biotec, Surrey, United Kingdom) was used to perform a Ficoll density gradient centrifugation in order to isolate mononuclear cells from blood samples. Cytospin and Fast Panoptic Staining (Panreac, Barcelona, Spain) allowed the analysis of these cells. Only preparations characterized by more than 90% of cells were considered for the analysis. Mononuclear cells were used to extract total RNA and to quantify the mRNA levels of PGC-1alpha and for miRNA quantification.

### Total oxyradical scavenging capacity assay (TOSCA)

Plasma antioxidant activity was assessed using the TOSC assay, whereby the antioxidant capacity of a molecule or biological liquid is quantified by its ability to inhibit ethylene formation compared with a control solution^58^. Set-up experiments were carried out to verify the correspondence with the ROS amount in plasma samples. Specifically, thermal homolysis of 20 mM 2,2′-azo-bis-amidinopropane (ABAP) at 35 °C in 100 mM potassium phosphate buffer, pH 7.4 was used to produce peroxyl radicals (ROO·) while hydroxyl radicals (OH·) were generated at 35 °C by the Fenton reaction (1.8 μM Fe^3+^, 3.6 μM EDTA, and 180 μM ascorbic acid in 100 mM potassium phosphate buffer, pH 7.4). 10 ml vials sealed with gas-tight Mininert^®^ valves (Supelco, Bellefonte, PA) were used to perform reactions with 0.2 mM of KMBA in a final volume of 1 ml. Gas Chromatographic analysis of 200 μl aliquots from the headspace of vials at timed intervals measured the Ethylene production. A Hewlett-Packard gas chromatograph (HP 6890 Series, Andoven, MA) with a Supelco SPB-1 capillary column (30 × 0.32 × 0.25 mm) and a flame ionization detector (FID) was used. Helium was used as the carrier gas (at a flow rate of 1 ml/min); a split ratio of 20:1 was used. TOSC values were quantified by the equation TOSC = 100 − (SA/CA × 100), where SA and CA are the integrated areas for the sample and control reaction, respectively. A TOSC value of 0 corresponds to a sample with no scavenging ability^59, 60^.

### Determination of the plasma concentrations of Nitrite and Nitrate concentrations, SIRT1 and PGC-1α

Plasma was stored at − 80 °C until the analysis of Nitrite and Nitrate (NOx) concentrations (which are considered stable end products of NO pathway), SIRT1, and PGC-1α. NOx determination was performed using a colorimetric assay kit (Cayman, Ann Arbor, MI, U.S.A.) based on the three-step Griess reaction^61^ and data are expressed as mM.

Plasma level of SIRT1 was detected by an enzyme-linked immunosorbent assay (ELISA) using a commercially available kit Human SIRT1 ELISA Kit—Invitrogen (thermofisher.com, Milan, Italy).

mRNA PGC-1α levels in blood samples (mononuclear cells) were determined using a Real-time quantitative PCR. Trizol™ (Invitrogen) was used to detect Total RNA according to the manufacturer’s instructions using a bioanalyzer RNA quality was evaluated and then quantified in a nanodrop. The ratio of absorbance of the RNA was at 260 nm and 280 nm of ≥ 1.8 while its integrity number was ≥ 8. A retro-transcribed cDNA with specific primers^62^ was used to detect mRNA expression levels of PGC-1α. The results are given as the number of times greater than the minimum value among all patients.

### MiRNA quantification

A reverse transcription real-time polymerase chain reaction (RT-qPCR) was used for miRNA quantification. The extracted RNA was used to transcribe cDNA (Qiagen, Hilden, Germany). Primers for miRNA sequences have been reported elsewhere^63,64^. The QIAgility instrument (Qiagen, Hilden, Germany) with an automated pipetting protocol performs cDNA steps and PCR setup. Rotor-Gene PCR cycling was executed following the instructions. The exponential increase in miRNA fluorescence determines the Cycle threshold (Ct). miRNAs were considered as detected when Ct values were lower than 33, otherwise they weren’t detected by GeneGlobe Data Analysis Center PCR software (Qiagen). ΔCt is the normalization of miRNA expression at baseline and post-training and it was calculated as previously described^63,64^. After this, the ΔΔCt was determined by subtracting baseline ΔCt values from post-training ΔCt values. 2 − ΔΔCt was the fold change.

### Statistical analysis

The results were expressed as mean value ± standard deviation (SD). Endothelium-dependent and endothelium-independent microvascular function were measured by peak skin flow responses to heating and to ischemia and reperfusion, measured in conventional PUs (perfusion units)^65^. Student’s t-test for non-paired data was used to compare basal HBF and FBF values obtained in ATL and in SED subjects. Analysis of variance (ANOVA) for repeated measures (Scheffe ´’s test for multiple comparison testing) was used to compare the skin blood flux response to heating or ischemia between ATL and SED. A mixed model analysis of covariance (ANCOVA) (group by time) was used to explore the relations between each of the skin blood flux values and the clinical or biochemical parameters investigated in ATL and SED subjects. Differences were considered significant at a *P* value of < 0.05. All statistical procedures were performed using the StatView program (Abacus Concepts, Inc., SAS Institute, Cary, NC)^66,67^.

### Ethical approval

The study was conducted in accordance with the Declaration of Helsinki, and approved by the the Ethics Committee (CTO, Clinical Trial Office) of Azienda Ospedaliero Universitaria Pisana (AOUP) (protocol code 35,105, approved on 13 June 2019).

### Informed consent

Informed consent was obtained from all subjects involved in the study.

## Conclusions

In conclusion, master ATL had a better microcirculatory function compared to sedentary controls as well as a higher plasma NO bioavailability and a greater antioxidant capacity against hydroxyl and peroxyl radicals; moreover, ATL exhibited increased transcriptional levels of the metabolism regulator PGC-1α and higher levels of SIRT1 and miRN29, with a consequent enhancement of those pathways involved in redox balance and cellular metabolism. Despite the advanced age of athletes involved in the present study, our data confirm that regular physical exercise activates intracellular pathways and epigenetic modifications affecting downstream redox homeostasis and the bioavailability of NO, ideally preventing endothelial cell senescence.

In interpreting our results, a few limitations should be underlined; besides the limited number of subjects, the mean age of the enrolled subjects was around 50 years, with a limited standard deviation. Future and interesting studies would analyze the presented parameters in older populations and in a wide range of age, in order to test the influence of age on these parameters. Finally, different validated methodology, including the strain gauge plethysmography, would be used to measure skin blood flow.

### Supplementary Information

Supplementary Information.

## Data Availability

All data generated or analyzed during this study are included in this published article (and its Supplementary Information files).
