# Supplementary material for: Regular exercise delays microvascular endothelial dysfunction by regulating antioxidant capacity and cellular metabolism
Source: Sci Rep. 2023 Oct 17;13:17671. doi: 10.1038/s41598-023-44928-4 (PMC10582030; doi:10.1038/s41598-023-44928-4)
Supplement: Supplementary file 1 — Supplementary Information. [file 41598_2023_44928_MOESM1_ESM.docx]

**Supplementary Tables**

|  |  | **Correlation** | **p-value** |
| --- | --- | --- | --- |
| HBF basal, HBF heating | | 0.524 | 0.0001 |
| HBF basal, HBF ischemia | | 0.310 | 0.0359 |
| HBF basal, FBF basal | | 0.969 | < 0.0001 |
| HBF basal, FBF heating | | 0.414 | 0.0039 |
| HBF basal, FBF ischemia | | 0.348 | 0.0172 |
| HBF heating, HBF ischemia | | 0.778 | < 0.0001 |
| HBF heating, FBF basal | | 0.507 | 0.0004 |
| HBF heating, FBF ischemia | | 0.780 | < 0.0001 |
| HBF ischemia, FBF basal | | 0.334 | 0.0279 |
| HBF ischemia, FBF heating | | 0.509 | 0.0002 |
| FBF basal, FBF heating | | 0.473 | 0.0011 |
| FBF basal, FBF ischemia | | 0.396 | 0.0081 |
| FBF heating, FBF ischemia | | 0.626 | < 0.0001 |

**Supplementary Table S1.** Correlation between clinical parameters; p values obtained for each correlation are reported in the respective columns. HBF (Hand Blood Flow); FBF (Foot Blood Flow).

|  | **Correlation** | **p-value** |
| --- | --- | --- |
| NOx (mM), miR29 | 0.216 | 0.0484 |
| NOx (mM), TOSC vs. peroxyl radicals | 0.428 | 0.0030 |
| NOx (mM), TOSC vs. hydroxyl radicals | 0.437 | 0.0021 |
| HBF basal, SIRT1 (ng/ml, plasma) | 0.330 | 0.0265 |
| HBF heating, SIRT1 (ng/ml, plasma) | 0.407 | 0.0052 |
| HBF heating, miR29 | 0.358 | 0.0141 |
| HBF heating, TOSC vs. peroxyl radicals | 0.747 | < 0.0001 |
| HBF heating, TOSC vs. hydroxyl radicals | 0.715 | < 0.0001 |
| HBF ischemia, SIRT1 (ng/ml, plasma) | 0.446 | 0.0019 |
| HBF ischemia, miR29 | 0.540 | < 0.0001 |
| HBF ischemia, PGC-1α mRNA | 0.421 | 0.0032 |
| HBF ischemia, TOSC vs. peroxyl radicals | 0.768 | < 0.0001 |
| HBF ischemia, TOSC vs. hydroxyl radicals | 0.673 | < 0.0001 |
| FBF basal, SIRT1 (ng/ml, plasma) | 0.372 | 0.0148 |
| FBF basal, TOSC vs. peroxyl radicals | 0.309 | 0.0461 |
| FBF basal, TOSC vs. hydroxyl radicals | 0.309 | 0.0435 |
| FBF heating, SIRT1 (ng/ml, plasma) | 0.428 | 0.0030 |
| FBF heating, miR29 | 0.370 | 0.0108 |
| FBF heating, TOSC vs. peroxyl radicals | 0.490 | 0.0005 |
| FBF heating, TOSC vs. hydroxyl radicals | 0.456 | 0.0013 |
| FBF ischemia, SIRT1 (ng/ml, plasma) | 0.511 | 0.003 |
| FBF ischemia, miR29 | 0.469 | 0.0009 |
| FBF ischemia, PGC-1α mRNA | 0.381 | 0.0086 |
| FBF ischemia, TOSC vs. peroxyl radicals | 0.760 | < 0.0001 |
| FBF ischemia, TOSC vs. hydroxyl radicals | 0.757 | < 0.0001 |

**Supplementary Table S2.** Correlation between clinical and biochemical parameters; p values obtained for each correlation are reported in the respective columns. BMI (Body Mass Index); NOx (Nitric Oxide); HBF (Hand Blood Flow); FBF (Foot Blood Flow); SIRT1 (Sirtuin 1); PGC-1α (PPAR-γ co-activator-1 α); TOSC (Total Oxyradical Scavenging Capacity).
